# Variant pathogenic prediction by locus variability: the importance of the current picture of evolution

**DOI:** 10.1038/s41431-021-01034-1

**Published:** 2022-01-26

**Authors:** José Luis Cabrera-Alarcon, Jorge García Martinez, José Antonio Enríquez, Fátima Sánchez-Cabo

**Affiliations:** 1 Centro Nacional de Investigaciones Cardiovasculares (CNIC), Melchor Fernandez Almagro 3, 28029 Madrid, Spain; 2 Data Analysis Unit, Instituto de Investigación Sanitaria, Hospital de la Princesa, Madrid, Spain; 3 Centro de Investigaciones Biomédicas en Red en Fragilidad y Envejecimiento Saludable (CIBERFES), Melchor Fernandez Almagro 3, 28029 Madrid, Spain

**Keywords:** Computational biology and bioinformatics, High-throughput screening

## Abstract

Accurate detection of pathogenic single nucleotide variants (SNVs) is a key challenge in whole exome and whole genome sequencing studies. To date, several *in silico* tools have been developed to predict deleterious variants from this type of data. However, these tools have limited power to detect new pathogenic variants, especially in non-coding regions. In this study, we evaluate the use of a new metric, the Shannon Entropy of Locus Variability (SELV), calculated as the Shannon entropy of the variant frequencies reported in genome-wide population studies at a given locus, as a new predictor of potentially pathogenic variants in non-coding nuclear and mitochondrial DNA and also in coding regions with a selective pressure other than that imposed by the genetic code, *e.g* splice-sites. For benchmarking, SELV was compared to predictors of pathogenicity in different genomic contexts. In nuclear non-coding DNA, SELV outperformed CDTS (AUC_SELV_ = 0.97 in ROC curve and PR-AUC_SELV_ = 0.96 in Precision-recall curve). For non-coding mitochondrial variants (AUC_SELV_ = 0.98 in ROC curve and PR-AUC_SELV_ = 1.00 in Precision-recall curve) SELV outperformed HmtVar. Moreover, SELV was compared against two state-of-the-art ensemble predictors of pathogenicity in splice-sites, ada-score, and rf-score, matching their overall performance both in ROC (AUC_SELV_ = 0.95) and Precision-recall curves (PR-AUC = 0.97), with the advantage that SELV can be easily calculated for every position in the genome, as opposite to ada-score and rf-score. Therefore, we suggest that the information about the observed genetic variability in a locus reported from large scale population studies could improve the prioritization of SNVs in splice-sites and in non-coding regions.

## Introduction

Whole Exome and Whole Genome Sequencing (WES, WGS) have revolutionized the way we study a range of genetic diseases. However, given the high degree of human variability, WES/WGS analysis renders a large number of variants, making it challenging to discriminate pathogenic from neutral variants. For this purpose, researchers have built several predictors to aid variant prioritization for the detection of deleterious variants.

To date, gnomAD represents the greatest effort to summarize human population genetic variability, including around 241 million variants detected in 125,748 WES and 15,708 WGS from unrelated individuals in v2.1 and 76,156 WGS from unrelated individuals in v3.1 [1]. Similarly, helixMTdb compiles human genetic variability in mitochondrial DNA from 196,554 individuals [2]. It is known that pathogenic variants are observed at low frequencies in the population and that they appear in regions with stronger selective pressure [3]. Therefore, it is interesting to study how the number and distribution of different variants present in a population at a given locus, might also provide relevant information about the pathogenicity of the variants.

Recent studies have modeled mutation probabilities based on mutation frequencies observed at 5 or 7-mers, highlighting the clear influence of neighboring nucleotides in the pathogenicity of a variant [4, 5]. However, the effect of nucleotide context may also be reflected in allele frequencies associated with the variants described for that specific position.

The genetic variability observed at a given locus in coding DNA might, however, be distorted due to the redundancy of the genetic code [6], resulting in the coexistence of synonymous and lethal single nucleotide variants (SNVs). However, locus variability could be useful for genomic positions in which the genetic code does not hold (non-coding regions) or the effect of the genetic code redundancy is diminished by the selective pressure imposed by an additional functionality, as the splicing in splice-sites. Unlike nuclear DNA, whose material is inherited from both parents and whose main source of variability is sister chromatid exchange, mitochondrial DNA is maternally inherited with a high mutation rate being its main source of variability [7]. Therefore, mitochondrial DNA has its own conservation path and population frequencies that may not resemble the behavior of nuclear DNA [8]. Hence, the specifics of mitochondrial DNA must be considered, in predictors of mitochondrial variant pathogenicity.

In this work, we hypothesize that the locus variability at a given genomic position observed in a population might be an indicator of the pathogenicity of the variants placed there. Hence, we propose a simple metric of locus variability based on the Shannon entropy (SELV) calculated over the population frequencies associated to a genomic position, in order to detect pathogenic SNVs within splice-sites and non-coding regions DNA. We compared the performance of SELV with established tools for variant prioritization and found that it adds highly valuable information for genomic positions that remain under-studied in genetically based diseases.

## Materials and methods

### Prediction of variants in splice-sites

**Fig. 1:**
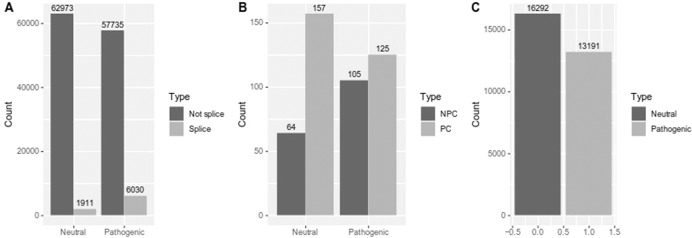
Distribution of neutral and pathogenic SNPs in analyzed data. Number of neutral/pathogenic in splice vs not-splice analysis (**A**), in dataset of mitochondrial SNVs (**B**), and in dataset used for benchmarking SELV in Non-coding variants (**C**). NPC: Non protein coding, PC: protein coding.

For the prediction of deleterious variants in splice-sites, we built a dataset with 131,002 unique variants (65,734 pathogenic, 65,268 neutral) retrieved from five independent benchmark data-sets *HumVar* [9]*, ExoVar* [10]*, VariBench* [11]*, predictSNP* [12] *and* SwissVar [13] and also variants selected from Clinvar [14], classified as benign or pathogenic variants. These 131,043 variants were classified as splice-site/not-splice-site (according to ensembl information for canonical transcript), resulting in 10,294 variants located in splice-sites and 120,708 not located in splice-sites. Then, we selected a subset of 7,941 out of these 10,294 variants for which there were pre-computed values for ada-score [15] and rf-score [15] in dbNSFP [16], to benchmark the use of the Shannon entropy locus variability metric (SELV) for the prediction of deleteriousness in splice-site SNVs. Neutral/pathogenic distribution in splice-site/Not-splice-site SNVs is depicted in Fig. 1A. The proportion of pathogenic variants in splice-sites was significantly larger than that for neutral variants (10.4% vs 3%, Fisher’s exact test*: p* < 0.001), reinforcing the relevance of accurate prediction of the pathogenicity of new variants located in splice-sites. Splice-site condition was annotated using Variant effect Predictor [17], which was also used to retrieve the ada-score and the rf-score values from dbNSFP. Moreover, phastCons [18] and phylop [19] conservation scores calculated on multiple sequence alignment from sequences of 100 species of vertebrates, was retrieved using UCSC table browser data retrieval tool [20].

### Prediction of variants in mitochondrial DNA

For the evaluation of SELV in mitochondrial DNA we first selected curated pathogenic variants plus variants with high frequency (Fq>0.01), considered to be neutral polymorphisms, reported in Mitomap [21]. In addition, mitochondrially encoded variants reported in *HumVar, ExoVar, VariBench, predictSNP*, *SwissVar* and *Clinvar* (only benign and pathogenic) were also chosen, resulting in a total amount of 451 SNVs. From them, 169 were located in non-coding regions (28 in control region, 108 in tRNA genes and 33 in rRNA genes) and 282 in protein-coding regions (Fig. 1B). From the pathogenic variants, 45.6% were located in non-protein-coding DNA, suggesting again the need for predictors of variant pathogenicity in non-coding mitochondrial DNA. These variants were annotated with HmtVar disease score [22], retrieving 336 annotated variants (112 non-coding; 224 protein-coding).

### Prediction of variants in non-coding nuclear DNA

For SELV assessment in non-coding nuclear DNA, pathogenic non-coding SNVs were obtained from those used for the context-dependent tolerance score (CDTS) assessment [23], while neutral non-coding SNVs were randomly selected from VariSNP [24]. Finally, the dataset contained a total of 29480 variants: 16,292 neutral SNVs and 13,188 pathogenic, Fig. 1C.

### SELV definition

At each locus, there is a number of variants reported in the population, either in gnomAD or in helixMTdb, with a specific frequency *F*_*i*_. Hence, for each locus there’s a vector of frequencies *(F*_*1*_*,…,F*_*n*_), where *F*_i_ is the frequency of variant i in the given locus, for each of the n variants at this place. Then SELV is defined as:

$$SELV = - \mathop {\sum}\limits_{i = 1}^n {F_i \times \log } (F_i)$$

SELV takes positive values. The higher the value of SELV, the more variability is observed for that genomic position.

In order to obtain the highest throughput for SELV calculation from gnomAD data, gnomAD v2.1 was used for splice site analysis, because it contains more splice site information, and v3.1 was used to assess pathogenicity in non-coding nuclear SNVs, because it contains a larger amount of WGS from unrelated individuals.

### Benchmarking

The performance of SELV in splice-site variants was compared with two state-of-the-art predictors, ada-score and rf-score, using AUC in receiver operating characteristic (ROC) curves and PR-AUC in precision recall (PR) curves. Then, AUC differences were tested for ROC curves, calculating the D-statistic, as described in pROC R-package, which was used for this purpose. ROC and PR curve analyses were performed using ROCR and precrec R-packages. In nuclear non-coding SNVs, SELV was compared to CDTS, while in non-coding mitochondrial SNVs, it was compared to HmtVar. In addition, SELV was also evaluated in protein-coding regions. In all three genetic contexts SELV performance was compared with the conservation scores phyloP and phastCons.

### SELV-based classification of variants of uncertain significance

SELV was used to reclassify variants of uncertain significance (VUS) obtained from Clinvar. For this, three different cut-off points for SELV were used, depending on whether the variants were splice-site, nuclear non-coding or mitochondrial non-coding SNVs, because the frequencies are obtained from different databases depending on the analysis. The cut-off values were calculated based on the Youden index.

SELV was also assessed to consider whether gnomAD ancestries can influence in SELV, (Supplementary document 1).

## Results

**Fig. 2:**
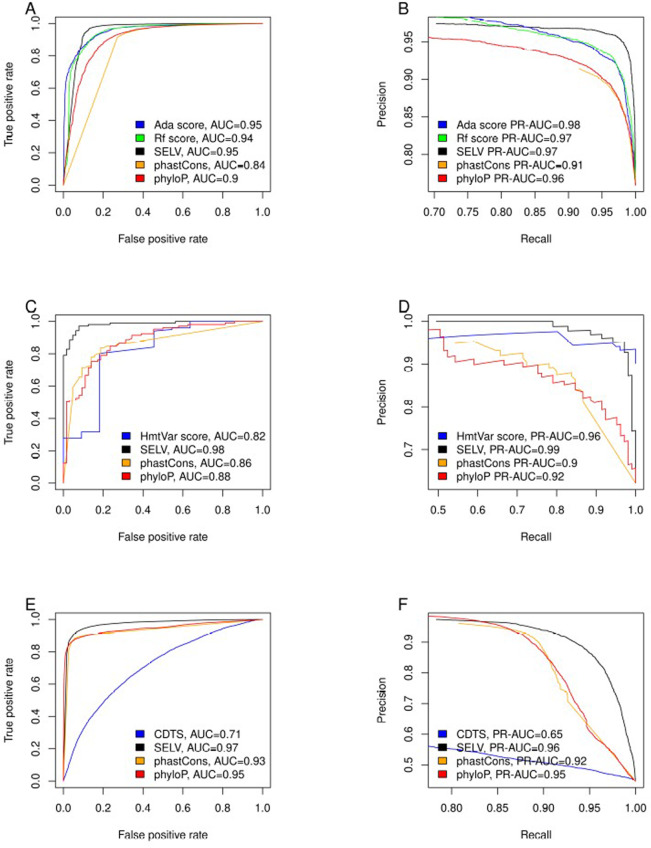
Performance of predictors compared to SELV. ROC (**A**) and PR curves (**B**) for SELV, ada score, rf score, phastCons, and phyloP conservation scores for deleteriousness in splice-site SNVs. ROC curves (**C**) and precision-recall curves (**D**) for SELV, HmtVar disease score phastCons, and phyloP conservation scores for pathogenic variant detection in mitochondrial non-coding SNVs. The performance of SELV, CDTS, phastCons and phyloP conservation scores for pathogenic variant detection in nuclear non-coding SNVs depicted as ROC (**E**) and PR curves (**F**). Abbreviations: ROC receiver operating characteristic, PR precision-recall, SELV Shannon entropy locus variability, CDTS context-dependent tolerance score, and SNV single nucleotide variants.

SELV distribution was significantly different in genomic positions spanning splice-site vs non-splice-sites (Kolmogorov–Smirnov: *D* = 0.186, *p* < 0.001). This result confirms that locus variability is a distinctive feature of variants located in splice sites vs coding regions of the genome. Hence, we tested the ability of SELV to predict the pathogenicity of variants located in splice-sites. SELV reached an AUC_SELV_ = 0.95, outperforming rf-score and showing similar behavior to ada-score (Fig. 2A). Regarding Precision-Recall performance, SELV achieved an PR-AUC_SELV_ = 0.97, matching rf-score results and being slightly surpassed by ada-score, (Fig. 2B).

For mitochondrial and nuclear non-coding SNVs, SELV presented the best behavior, both in ROC curves and PR curve (Fig. 2C–F).

All these differences were statistically significant, except for ada-score (Table 1). However, SELV can be calculated for every genomic position, while ada-score is only available for a limited number of locus, making SELV more usable.

**Table 1:** Comparison of the area under the receiving operative characteristic curve between different considered predictors and SELV, both in splice-sites and non-coding mitochondrial DNA.

Splice-site SNVs		AUC_SELV_ = 0.95
	AUC_rf_score_ = 0.94	*D* = 2.02, *p* < 0.05
	AUC_ada_score_ = 0.95	*D* = −0.35, *p* = 0.7232
	AUC_phastCons_ = 0.84	*D* = 19.12, *p* < 0.001
	AUC_phyloP_ = 0.9	*D* = 9.10, *p* < 0.001
Non-coding mitochondrial SNVs		AUC_SELV_ = 0.98
	AUC_HmtVar_ = 0.82	*D* = 2.34, *p* < 0.05
	AUC_phastCons_ = 0.86	*D* = 4.31, *p* < 0.001
	AUC_phyloP_ = 0.88	*D* = 3.99, *p* < 0.001
Non-coding nuclear SNVs		AUC_SELV_ = 0.97
	AUC_CDTS_ = 0.71	*D* = 83.59, *p* < 0.001
	AUC_phastCons_ = 0.93	*D* = 21.57, *p* < 0.001
	AUC_phyloP_ = 0.95	*D* = 14.91, *p* < 0.001

In all three analysis carried out, SELV outperformed phyloP and phastCons in both AUC and PR-AUC values. However, SELV performed poorly for protein coding variants, in both mitochondria (AUC_SELV_ = 0.68) and nuclear not-splice-site protein coding regions (AUC = 0.86).

Importantly, SELV was able to reclassify 62521 VUS retrieved from ClinVar: 2,005 SNVs in splice-sites, 60356 in non-coding nuclear regions, and 160 in non-coding mitochondrial DNA. In the splice-site regions, SELV was able to identify pathogenic variants with a 0.97 sensitivity and 0.87 specificity (threshold = 0.001), retrieving 90 neutral and 1915 pathogenic SNVs. In mitochondrial non-coding regions, SELV predicted pathogenicity with the same sensitivity but higher specificity, 0.92 (threshold = 0.009), yielding two neutral and 158 pathogenic variants. Finally, in non-coding nuclear DNA, the sensitivity was slightly worst (0.91) than in the previous genetic contexts, but the specificity increased up to 0.94 (threshold = 0.0002), resulting in the reclassification of VUS as 28,760 neutral and 31,596 pathogenic SNVs.

The data-sets used in the benchmarking and Clinvar’s reclassified VUS tables are listed in supplementary tables 1 and 2.

## Discussion

Our results confirm that locus variability is an important feature in genomic regions for which the genetic code redundancies are not relevant, eg. splice-sites. For these regions, SELV outperformed conservation-based scores and showed a similar performance to scores specific to splice-sites, such as ada-score and rf-score. However, ada-score and rf-score are machine learning ensemble scores that combine several predictors [15] and are only available for certain genomic positions, while SELV can be easily computed for each position in the genome, providing a great advantage over more sophisticated tools and without the loss of performance.

In the mitochondrial genome, the vast majority of predictors are focused in protein-coding variants with the significant exception of HmtVar. Considering Cambridge reference sequence for human mitochondrial DNA, non-coding DNA represents around 31% of the mitochondrial genome, pointing out the importance of developing a predictor oriented to non-coding regions. Regarding our results in non-coding sequences in mitochondria, it seems that SELV shows a straightforward relationship with deleteriousness and might be an interesting predictor, outperforming some widely used predictors such as HmtVar.

CDTS is an interesting model that quantifies the degree of tolerance of a region to mutate, taking into account surrounding sequences [23]. In spite of this interesting approach, SELV outperformed the results of CDTS for pathogenic SNVs detection in nuclear non-coding regions. Similarly, SELV was more accurate and precise than traditional conservation scores, in both nuclear and mitochondrial non-coding regions.

Regarding the behavior of SELV according to the different gnomAD populations considered (Supplementary document 1), generally, the reduction in population size leads to an increase in the number of false positives. It could be that the analyzed number of individuals per population is not enough to gather population variability. Nevertheless, the use of SELV by population could be an interesting approach, once the number of sampled subjects is increased.

All in all, SELV is a very accurate predictor of pathogenic variants in regions that escape the effect of the redundancy of the genetic code. It may represent an option to evaluate SNVs placed in non-coding regions, which represent the vast majority of the genome (>98%). Thanks to its simplicity, it can be easily incorporated into pipelines for variant prioritization, reducing the number of SNVs classified as variants of uncertain significance in mendelian or mitochondrial diseases.

## Supplementary information

Supplementary files description

Supplemntary document 1

Supplementary table 1

Supplementary table 2

## Data Availability

VariBench, http://structure.bmc.lu.se/VariBench/. Clinvar, https://www.ncbi.nlm.nih.gov/clinvar/. Mitomap, https://www.mitomap.org/MITOMAP. GnomAD, https://gnomad.broadinstitute.org/. VEP, http://grch37.ensembl.org/Homo_sapiens/Tools/VEP. VariSNP, http://structure.bmc.lu.se/VariSNP/.
