# Supplementary material for: Variant pathogenic prediction by locus variability: the importance of the current picture of evolution
Source: Eur J Hum Genet. 2022 Jan 26;30(5):555–9. doi: 10.1038/s41431-021-01034-1 (PMC9091277; doi:10.1038/s41431-021-01034-1)
Supplement: Supplementary file 1 — Supplementary files description [file 41431_2021_1034_MOESM1_ESM.docx]

**Description of supplementary files:**

**File Name:** Supplementary Document 1

**Description:** Analysis of the influence of gnomAD ancestry on SELV performance.

**File Name:** Supplementary table 1

**Description:** Data-sets used to evaluate SELV (separated in different sheets). These data-sets contain the following fields:

1. HGVS: The name of the variant following HGVS nomenclature.
2. CHROM: The chromosome where the variant is placed.
3. POS: Genomic coordinate of the SNVs.
4. REF: Reference nucleotide.
5. ALT: Nucleotide substitution the represents the variant.
6. Label: Class label information (0 = benign and 1 = pathogenic).
7. Predictors used in the comparative analysis:
   1. In data-set called Benchmark.Non.coding.nuclear.SNVs:
      - CDTS (context-dependent tolerance score).
      - Phastcons
      - phyloP
   2. In data-set called Benchmark.splice.site.SNVs:
      - ada_score
      - rf_score
      - Phastcons
      - phyloP
   3. In data-set called Benchmark.Non.coding.MT.SNVs:
      - HmtVar_disease_score
      - Phastcons
      - phyloP
8. SELV (Shannon Entropy Locus Variability)

In data-sets Benchmark.splice.site.SNVs and Benchmark.Non.coding.nuclear.SNVs there are additional columns with SELV values computed for gnomAD populations:

1. SELV.nfe (SELV computed in Non-Finnish European).
2. SELV.fin (SELV computed in Finnish).
3. SELV.asj (SELV computed in Ashkenazi Jewish).
4. SELV.afr (SELV computed in African/African American).
5. SELV.amr (SELV computed in Latino/Admixed American).
6. SELV.ami (SELV computed in Amish).
7. SELV.sas (SELV computed in South Asian).
8. SELV.eas (SELV computed in East Asian).
9. SELV.oth (SELV computed in Other populations).

IMPORTANT: In the Benchmark.Non.coding.nuclear.SNVs data-set SELV is computed considering the allele frequencies of gnomAD v3.1.2, in Benchmark.splice.site.SNVs all SELV values are calculated on the allele frequencies of 125,748 exomes in gnomAD v2.1.1 and in Benchmark.Non.coding.MT.SNVs SELV is estimated from the HelixMTdb data.

Variants for Non-coding SNVs analysis were collected in October 2021, remaining data were gathered in September 2020.

**File Name:** Supplementary table 2

**Description:** SELV-based classification of variants of uncertain significance from Clinvar, separated in different sheets considering the analysis (mitochondrial Non-coding SNVs, nuclear Non-coding SNVs and Splice site SNVs). These data-sets contain the following fields:

1. HGVS: The name of the variant following HGVS nomenclature.
2. CHROM: The chromosome where the variant is placed.
3. POS: Genomic coordinate of the SNVs.
4. REF: Reference nucleotide.
5. ALT: Nucleotide substitution the represents the variant.
6. SELV (Shannon Entropy Locus Variability).
7. Label: Class label information (0 = benign and 1 = pathogenic).

Variants of uncertain significance were collected in October 2021.
