# Supplementary material for: Variant pathogenic prediction by locus variability: the importance of the current picture of evolution
Source: Eur J Hum Genet. 2022 Jan 26;30(5):555–9. doi: 10.1038/s41431-021-01034-1 (PMC9091277; doi:10.1038/s41431-021-01034-1)
Supplement: Supplementary file 2 — Supplemntary document 1 [file 41431_2021_1034_MOESM2_ESM.docx]

SELV is a position specific statistic that is calculated as the Shannon entropy of the frequencies of the variants described for that position, in gnomAD[1] or helixMTdb[2]. As allele frequencies may change depending on the studied population, SELV was evaluated in order to consider whether gnomAD ancestries can influence in its throughput.

***Analysis of the influence of gnomAD ancestry on SELV performance***

To assess whether ancestry consideration can influence SELV for nuclear (splice-site and non-coding) SNVs, the results were summarized as the effect on ROC and PR curves. In this section, we only consider the ancestry present in both gnomAD v2.1 and v3.1. We then calculate the SELV using population-specific frequencies.

In all populations the AUC and PR-AUC decreased compared to the overall SELV value, table 1. In addition, the AUC and PR-AUC values worsen in the analysis of non-coding regions compared with the splice site analysis, which is based on a larger amount of data. In general, the performance of SELV across different populations (especially for PR-AUC) worsens as the number of individuals studied decreases.

Regarding to the behavior of SELV according to the different populations considered from gnomAD, generally the reduction in population size leads to an increase in the number of false positives. It could be that analyzed number of individuals by population is not enough to gather population’s variability. Nevertheless, the use of SELV by population could be interesting approach, once the amount of sampled subjects is increased.

Regarding the analysis of the influence of gnomAD ancestry on SELV, the AUC and PR-AUC decreased in all populations compared to the overall SELV value (Table 1), mainly due to the smaller sample size. In addition, the AUC and PR-AUC values worsen in the analysis of non-coding regions compared with the splice site analysis, which is based on a larger amount of data. In general, the performance of SELV across different populations (especially for PR-AUC) worsens as the number of individuals studied decreases. In these populations the effective size may be insufficient to capture the heterogeneity linked to a specific genomic position.

Table 1, SELV performance in pathogenic SNVs detection calculated for specific populations.

| **Population** | **Splice-site SNVs** | | | | | **Nuclear Non-coding SNVs** | | | | |
| --- | --- | --- | --- | --- | --- | --- | --- | --- | --- | --- |
|  | **Size** | **AUC** | **AUC**  **_(All)*_** | **PR-AUC** | **PR-AUC**  **_(All)*_** | **Size** | **AUC** | **AUC**  **_(All)*_** | **PR-AUC** | **PR-AUC**  **_(All)*_** |
| **Non Finnish European** | 64,603 | 0.89 | 0.95 | 0.94 | 0.97 | 34,029 | 0.82 | 0.97 | 0.7 | 0.96 |
| **Finnish** | 12,562 | 0.76 |  | 0.86 |  | 5,316 | 0.65 |  | 0.53 |  |
| **African &**  **African American** | 12,487 | 0.91 |  | 0.94 |  | 20,744 | 0.89 |  | 0.8 |  |
| **Latino &**  **Admixed American** | 17,720 | 0.92 |  | 0.95 |  | 7,647 | 0.86 |  | 0.75 |  |
| **Ashkenazi Jewish** | 5,185 | 0.77 |  | 0.86 |  | 1,736 | 0.65 |  | 0.53 |  |
| **South Asian** | 15,308 | 0.85 |  | 0.91 |  | 2,419 | 0.77 |  | 0.63 |  |
| **East Asian** | 9,977 | 0.76 |  | 0.86 |  | 2,604 | 0.68 |  | 0.55 |  |
| **Other** | 3,614 | 0.9 |  | 0.94 |  | 1,047 | 0.82 |  | 0.7 |  |
| *SELV computed considering all data.  Abbreviations: area under the receiver operating characteristic curve (AUC), area under the precision recal curve (PR-AUC), single nucleotide variants (SNVs). | | | | | | | | | | |
